# Differential regulation of tetramerization of the AMPA receptor glutamate–gated ion channel by auxiliary subunits

**DOI:** 10.1016/j.jbc.2023.105227

**Published:** 2023-09-09

**Authors:** Noele Certain, Quan Gan, Joseph Bennett, Helen Hsieh, Lonnie P. Wollmuth

**Affiliations:** 1 Graduate Program in Molecular and Cellular Pharmacology, Stony Brook University, Stony Brook, New York, USA; 2 Graduate Program in Neuroscience, Stony Brook University, Stony Brook, New York, USA; 3 Department of Neurobiology & Behavior, Stony Brook University, Stony Brook, New York, USA; 4 Department of Surgery, Renaissance School of Medicine, Stony Brook University, Stony Brook, New York, USA; 5 Center for Nervous System Disorders, Stony Brook University, Stony Brook, New York, USA; 6 Department of Biochemistry & Cell Biology, Stony Brook University, Stony Brook, New York, USA

**Keywords:** AMPAR, CNIH, TARP, GSG1-L, synaptic transmission, ER, membrane trafficking

## Abstract

α-amino-3-hydroxy-5-methyl-4-isoxazolepropionic acid receptor (AMPAR) auxiliary subunits are specialized, nontransient binding partners of AMPARs that modulate AMPAR channel gating properties and pharmacology, as well as their biogenesis and trafficking. The most well-characterized families of auxiliary subunits are transmembrane AMPAR regulatory proteins (TARPs), cornichon homologs (CNIHs), and the more recently discovered GSG1-L. These auxiliary subunits can promote or reduce surface expression of AMPARs (composed of GluA1-4 subunits) in neurons, thereby impacting their functional role in membrane signaling. Here, we show that CNIH-2 enhances the tetramerization of WT and mutant AMPARs, presumably by increasing the overall stability of the tetrameric complex, an effect that is mainly mediated by interactions with the transmembrane domain of the receptor. We also find CNIH-2 and CNIH-3 show receptor subunit-specific actions in this regard with CNIH-2 enhancing both GluA1 and GluA2 tetramerization, whereas CNIH-3 only weakly enhances GluA1 tetramerization. These results are consistent with the proposed role of CNIHs as endoplasmic reticulum cargo transporters for AMPARs. In contrast, TARP γ-2, TARP γ-8, and GSG1-L have no or negligible effect on AMPAR tetramerization. On the other hand, TARP γ-2 can enhance receptor tetramerization but only when directly fused with the receptor at a maximal stoichiometry. Notably, surface expression of functional AMPARs was enhanced by CNIH-2 to a greater extent than TARP γ-2, suggesting that this distinction aids in maturation and membrane expression. These experiments define a functional distinction between CNIHs and other auxiliary subunits in the regulation of AMPAR biogenesis.

The majority of fast excitatory neurotransmission in the nervous system relies on α-amino-3-hydroxy-5-methyl-4-isoxazolepropionic acid receptor (AMPARs) (1, 2). AMPARs are glutamate-gated ion channels that are regulated by the composition of the AMPAR macromolecular complex. The core of the complex is formed by pore-forming AMPAR subunits (GluA1-4). Functional properties of the core ion channel vary in a subunit-specific manner as well as by various posttranslational modifications (*e.g.*, RNA editing, glycosylation) (3, 4, 5, 6). In addition, this core AMPAR complex is typically associated with membrane-spanning auxiliary subunits (7, 8, 9, 10). Although these auxiliary subunits are not directly involved in the formation of the ion channel, they influence synaptic transmission by modulating AMPAR gating kinetics, ion permeation, and channel block as well as pharmacological properties (7, 11, 12, 13). AMPAR auxiliary subunits also regulate surface expression and distribution of receptors (13, 14, 15). Auxiliary subunits associate with AMPARs in a neuronal and subunit-specific–dependent manner, which contributes greatly to the functional diversity of AMPARs in the mammalian central nervous system (16, 17, 18, 19).

Transmembrane AMPAR regulatory proteins (TARPs) and cornichon homologs (CNIHs) are the most abundant AMPAR auxiliary subunits in the brain (17). Similar to TARPs, (CNIH-2 and CNIH-3 enhance AMPAR channel conductance and slow receptor deactivation and desensitization (20, 21, 22) despite a distinctive evolutionary origin. CNIH-2 also shows enrichment in both the endoplasmic reticulum (ER) and synaptic compartments (23). CNIHs function as ER cargo exporters, mediating AMPAR maturation and translocation from the ER and Golgi complex to the surface membrane (20, 24, 25). Neurons lacking CNIH-2/CNIH-3 show reduced synaptic AMPAR activity (26). Knockdown of CNIH-2 results in the retention of AMPARs and therefore disruption of synaptic functions (26).

Stargazin (TARP γ-2), the prototypical member of the TARPs family, is the most extensively studied AMPAR auxiliary subunit. TARP γ-2 increases AMPAR agonist potency and efficacy, channel conductance, and slows AMPAR deactivation and desensitization (27, 28, 29). In addition to the impact of TARP γ-2 on receptor functions, TARP γ-2 also increases AMPAR surface expression and participates in synaptic targeting and receptor endocytosis, which underlies its implication in various forms of synaptic plasticity (30, 31, 32). Under certain conditions such as ER stress, TARP γ-2 promotes the delivery of AMPARs to the surface but in a less efficient manner compared to CNIH-2 (23, 33, 34). TARP γ-2 plays an essential role in receptor surface trafficking (35, 36) but may come into play at later stages of receptor maturation (34).

TARP γ-8, another prominent TARP preferentially expressed in the hippocampus, regulates AMPAR trafficking and synaptic density (37, 38). The knockdown of TARP γ-8 in primary hippocampal neurons leads to gross reduction in surface AMPAR expression (39). GSG1-L is a more recently identified AMPAR auxiliary subunit, which is structurally similar to the TARP family (40, 41). GSG1-L reduces AMPAR surface expression by facilitating the endocytosis of surface AMPARs (42, 43). Although auxiliary subunits are involved in receptor trafficking, mechanisms involved in these processes are unclear.

In the present study, we explored the role of auxiliary subunits in regulating AMPAR tetramerization, an early step in receptor maturation (4, 44, 45). We find that CNIH-2 and CNIH-3 enhance WT AMPAR tetramerization by increasing the number of available tetramers. This effect is likely mediated by interactions between CNIHs and the transmembrane domain (TMD) of AMPARs. In contrast, other prominent auxiliary subunits including TARP γ-2 and γ-8 and GSG1-L have no notable effect on AMPAR tetramerization. We therefore conclude that CNIHs differ in their ability to modulate AMPAR tetramerization based on the stage of involvement with AMPAR biogenesis in comparison to other AMPAR auxiliary subunits.

## Results

To address the role of AMPAR auxiliary subunits in receptor tetramerization, we expressed AMPARs either alone or in the presence of different auxiliary subunits. We used blue native-PAGE (BN-PAGE) to evaluate the oligomeric states of AMPARs in whole-cell lysates. Initially, we assayed GluA1 tetramerization.

### CNIHs uniquely promote GluA1 tetramerization

**Figure 1:**
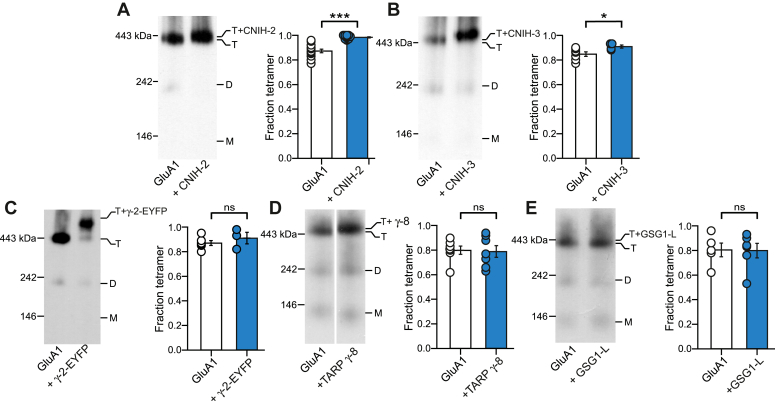
**Cornichon homologs promote assembly of GluA1 tetramers.***A–E,* BN-PAGE (*left panels*) and normalized tetramer fractions (*right panels*) of GluA1 expressed alone or with auxiliary subunits CNIH-2 (*A*), CNIH-3 (*B*), TARP γ-2-EYFP (γ-2-EYFP) (*C*), TARP γ-8 (*D*), or GSG1-L (*E*). Normalized tetramers fraction in this figure and all subsequent figures are shown as mean ± SEM. Constructs were expressed in HEK 293 cells. For BN-PAGE, positions of molecular mass markers are labeled on the *left* (see Experimental procedures). Oligomeric states of the detected bands were estimated by molecular mass and indicated as T (tetramers), D (dimers), or M (monomers). The fraction of tetramer was calculated from the cumulative intensities of the tetramer band(s), which included nonshifted and shifted bands, divided by the cumulative intensities of all oligomers (monomer, dimers, and tetramers) (see Experimental procedures). For bar plots, *circles* represent individual values. Number of samples (*left to right*) and significance (*t* test) of tetramer fraction relative to respective control (*white bar*): 13, 10, *p* < 0.001 (*A*); 7, 4, *p* = 0.03 (*B*); 6, 3, *p* = 0.52 (*C*); 7, 7, *p* = 0.84 (*D*); and 5, 6, *p* = 0.95 (*E*). In plots, significance is indicated (*∗p* < 0.05 or *∗∗∗p* < 0.001). BN-PAGE, blue native–PAGE; CNIH, cornichon; HEK, human embryonic kidney; *ns*, not significant; TARP, transmembrane AMPAR regulatory protein.

GluA1 expressed alone readily oligomerizes, forming predominantly tetramers with a small albeit notable dimer band and generally no monomer bands (Fig. 1, *A*–*E*, left panels) (4, 44, 46, 47, 48). In the presence of CNIH-2, GluA1 tetramerization was nearly complete, with negligible amount of dimer remaining (Fig. 1*A*, left panel). To quantify these results, we derived the tetramer fraction from cumulative oligomeric band intensities (see Experimental procedures). Although GluA1 alone showed a high tetramer fraction, when coexpressed with CNIH-2, the protein fraction was now almost completely tetrameric (Fig. 1*A*, right panel).

Within the CNIH homologs, CNIH-2 shares a unique sequence with CNIH-3, which carries specificity for binding to AMPARs (20, 40, 49). When coexpressed with homomeric GluA1 (Fig. 1*B*), CNIH-3 led to a significant increase in the tetramer fraction, like CNIH-2, although a small dimer population remained. In contrast to CNIHs, other AMPAR auxiliary subunits including TARP γ-2 (Fig. 1*C*), TARP γ-8 (Fig. 1*D*), and GSG1-L (Fig. 1*E*) showed no significant effect on receptor tetramerization. Overall, these results suggest that CNIHs have the unique function to enhance AMPAR tetramerization.

### CNIHs can rescue disrupted AMPAR tetramerization

Interactions between the M4 transmembrane helices and the ion channel core (M1-M3) are required to assemble a functional AMPAR (44, 48, 50, 51). Within the M4 segment are key residues, the “VVLGAVE” motif, that directly interact with the ion channel core (52). Amino acid substitutions at these positions lead to altered receptor tetramerization (44, 50).

**Figure 2:**
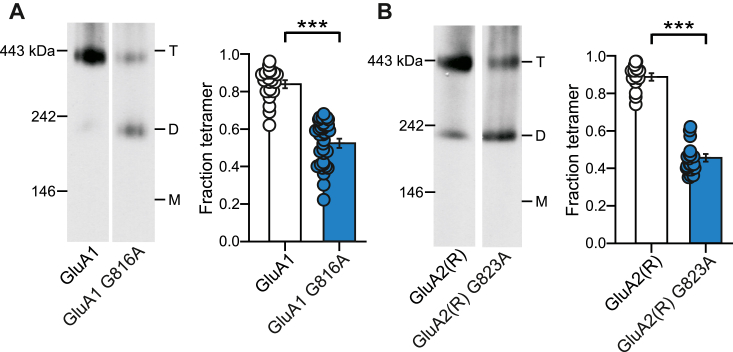
**Altered VVLGAVE motif disrupts AMPAR tetramerization**. *A* and *B,* BN-PAGE (*left panels*) and normalized tetramer fractions (*right panels*) of GluA1 compared to GluA1 G816A (*A*) or GluA2(R) compared to GluA2(R) G823A (*B*). Number of samples (*left* to *right*) and significance (*t* test) of tetramer fraction relative to control: 18, 25, *p* < 0.0001 (*A*); 13,16, *p* < 0.0001. In plots, significance is indicated (∗*p* < 0.05 or ∗∗∗*p* < 0.001)*.* AMPAR, α-amino-3-hydroxy-5-methyl-4-isoxazolepropionic acid receptor; BN-PAGE, blue native–PAGE; *ns*, not significant.

**Figure 3:**
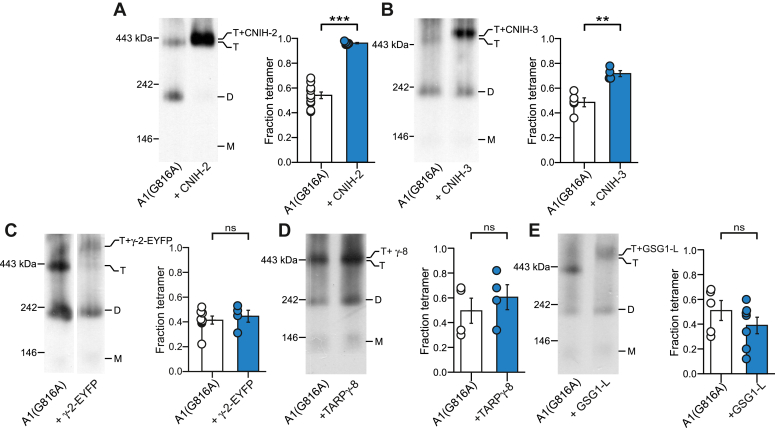
**Cornichon homologs can rescue deficits in AMPAR tetramerization.***A–E,* BN-PAGE (*left panels*) and normalized tetramer fractions (*right panels*) of GluA1(G816A) expressed alone or with CNIH-2 (*A*), CNIH-3 (*B*), TARP γ-2-EYFP (γ-2-EYFP) (*C*), TARP γ-8 (*D*), or GSG1-L (E). G816A is a mutation in the M4 VVLGAVE face that attenuates receptor tetramerization. A comparison of WT and G816A when run on the same day: GluA1, 0.84 ± 0.02, n =18; GluA1(G816A), 0.52 ± 0.02, n = 25. Significance (*t* test), *p < 0.001*. Number of samples (*left* to *right*) and significance (*t* test) of tetramer fraction relative to respective control (*white bar*): 11, 6, *p* < 0.001 (*A*); 5, 4, *p* = 0.0014 (*B*); 8, 4, *p* = 0.61 (*C*); 4, 4, *p* = 0.46 (*D*); and 5, 7, *p* = 0.28 (*E*). In plots, significance is indicated (*∗∗p* < 0.01 or *∗∗∗p* < 0.001) AMPAR, α-amino-3-hydroxy-5-methyl-4-isoxazolepropionic acid receptor; BN-PAGE, blue native–PAGE; CNIH, cornichon; *ns*, not significant; TARP, transmembrane AMPAR regulatory protein.

To further test whether CNIHs promote the tetramerization process in comparison to other AMPAR auxiliary subunits, we coexpressed auxiliary subunits with GluA1 AMPARs containing a single-point mutation, a glycine (G) to alanine (A), in the “VVLGAVE” motif. Despite the subtle difference in the side chain, G816A in GluA1 (Fig. 2*A*) or G823A in the edited form of GluA2, GluA2(R) (Fig. 2*B*) attenuates tetramer formation to about 50% assayed using either BN-PAGE (Fig. 2) or fluorescent size–exclusion chromatography (50). Notably, coexpression with CNIH-2 fully rescued the tetramerization deficit of GluA1(G816A) to WT levels (Fig. 3*A*). CNIH-3 also rescued the GluA1(G816A) tetramerization deficit, although to a lesser extent (Fig. 3*B*). In contrast, and as observed for WT GluA1, coexpression of TARP γ-2 (Fig. 3*C*), TARP γ-8 (Fig. 3*D*), or GSG1-L (Fig. 3*E*) with GluA1(G816A) had no significant effect on the tetramer fraction. In summary, CNIHs but most notably CNIH-2 has a strong effect on the assembly process of GluA1, whereas other AMPAR auxiliary subunits do not.

### CNIH-2 distinctively enhances GluA2(R) tetramerization

Like GluA1, the GluA2 AMPAR subunit is prominently expressed throughout the brain and interacts with a variety of auxiliary subunits (17, 53). GluA2 exists exclusively in an edited form, indicated as GluA2(R), where the amino acid glutamine (Q) is replaced with arginine (R) in the mature protein (2). We therefore tested the effects of auxiliary subunits on GluA2(R) tetramerization.

**Figure 4:**
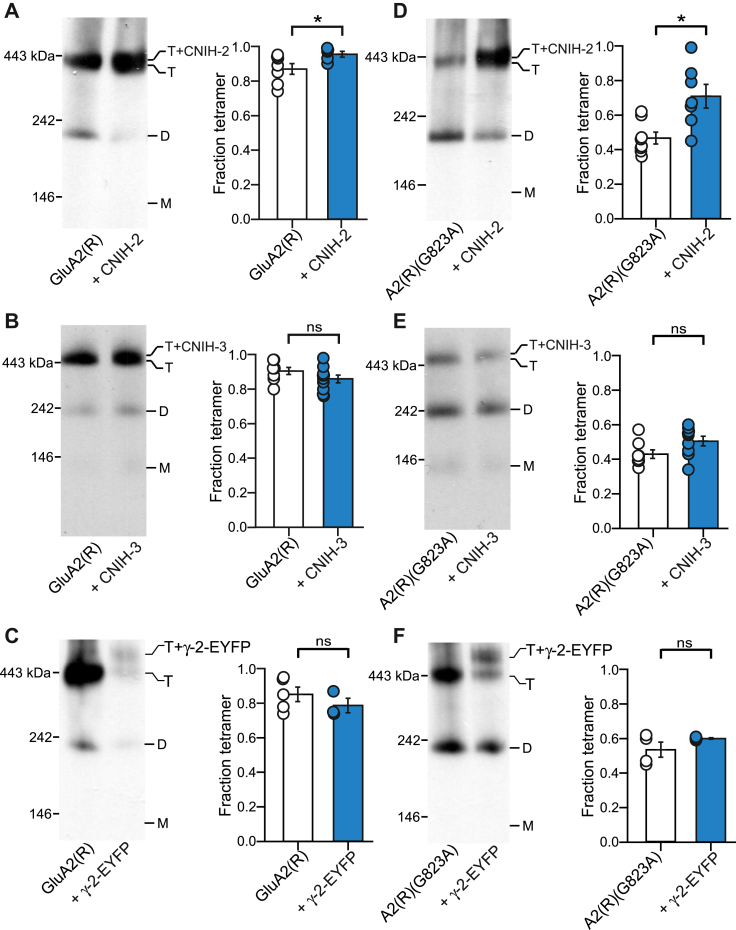
**Cornichon homologs differentially regulate GluA2 assembly.***A–F,* BN-PAGE (*left panels*) and normalized tetramer fractions (*right panels*) of GluA2(R) (*A–C*) or GluA2(R)(G823A) (*D–F*) expressed alone or with indicated auxiliary subunits. G823A is a mutation in the M4 VVLGAVE face that attenuates receptor tetramerization. A comparison of WT and G823A when run on the same day: GluA2(R), 0.89 ± 0.02, n =15; GluA2(R)(G823A), 0.45 ± 0.02, n = 16. Significance (*t* test), *p* < 0.001. Number of samples (*left* to *right*) and significance (*t* test) of tetramer fraction relative to respective control (*white bar*): 7, 5, *p* = 0.042 (*A*); 8, 10, *p* = 0.14 (*B*); 5, 3, *p* = 0.31 (*C*); 8, 7, *p* = 0.011 (*D*); 8, 9, *p* = 0.068 (*E*); and 4, 4, *p* = 0.22 (*F*). In plots, significance is indicated (*∗p* < 0.05). BN-PAGE, blue native–PAGE; CNIH, cornichon homolog; *ns*, not significant.

Like GluA1, homomeric GluA2(R) efficiently forms tetramer (Fig. 4, *A*–*C*) (4, 54). When coexpressed with CNIH-2, WT GluA2(R) showed enhanced tetramerization similar to the effects on GluA1 (Fig. 4*A*). In contrast, coexpression of CNIH-3 with GluA2(R) showed no change to the tetramer fraction (Fig. 4*B*), suggesting a differential effect between CNIH-2 and CNIH-3 on GluA2(R) tetramerization. To further verify these differential effects, we assayed the G-to-A substitution (VVLGAVE) in GluA2(R) that attenuates tetramerization (Fig. 2). Consistent with what is observed for WT GluA2(R), the tetramerization process for GluA2(R)(G823A) was recovered by coexpression with CNIH-2 (Fig. 4*D*), but not with CNIH-3 (Fig. 4*E*). Additionally, TARP γ-2 had no significant effect on GluA2(R) tetramerization, either for WT GluA2(R) (Fig. 4*C*) or for GluA2(R)(G823A) (Fig. 4*F*).

**Figure 5:**
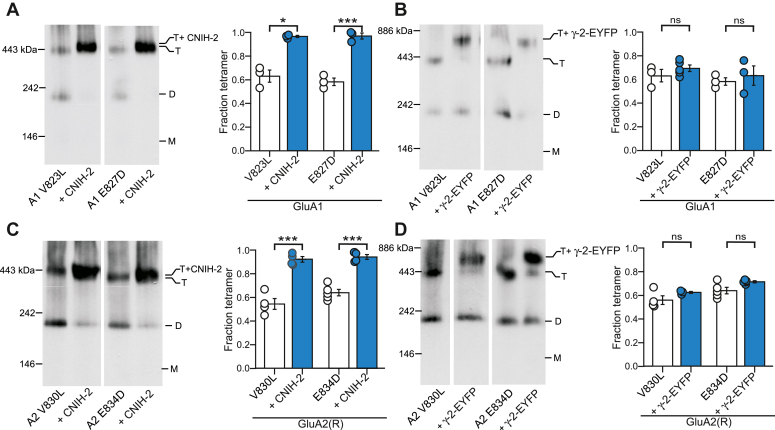
**Rescue of AMPAR tetramerization by CNIH-2 is independent of the position altered in the VVLGAVE motif.***A–D,* BN-PAGE (*left panels*) and normalized tetramer fractions (*right panels*) for single substitutions in the VVLGAVE motif in GluA1 (*A* and *B*) or GluA2(R) (*C* and *D*) expressed alone or with indicated auxiliary subunits. Constructs that disrupt tetramerization include the following: GluA1(V823L) (*A*), GluA1(E827D) (*B*), GluA2(R)(V830L) (*C*), and GluA2(R)(E834D) (*D*). Number of samples (*left* to *right*) and significance (*t* test) of tetramer fraction relative to respective control (*white bar*): for CNIH-2: 3, 3, *p* < 0.05 and for γ-2: 3, 3 *p* = 0.62 (*A*); for CNIH-2: 3, 3, *p* < 0.001 and for g-2: 3, 3, *p* = 0.62 (*B*); for CNIH-2: 4, 4, *p* < 0.001 and for g-2: 4, 3, *p* = 0.16 (*C*); for CNIH-2: 5, 4, *p* < 0.001 and for γ-2: 5, 4, *p* = 0.06 (*D*). In plots, significance is indicated (∗*p* < 0.05, *∗∗∗p* < 0.001). AMPAR, α-amino-3-hydroxy-5-methyl-4-isoxazolepropionic acid receptor; BN-PAGE, blue native–PAGE; CNIH, cornichon homolog; *ns*, not significant.

The results with the G-to-A substitution in the “VVLGAVE” face strongly supports the differential role of CNIH-2 in enhancing GluA1 (Fig. 3) and GluA2(R) (Fig. 4) tetramerization. However, it is possible that a single substitution within the “VVLGAVE” motif can specifically disrupt the function of TARP γ-2, but not that of CNIHs. We therefore tested other subtle mutations in the “VVLGAVE” motif where the side chain replacement was similar to the endogenous residues: in GluA1(V823L) and (E827D); and in GluA2(R)(V830L) and (E834D). These substitutions in GluA1 attenuate tetramerization (50). Based on BN-PAGE, these substitutions produce moderate tetramerization deficits that were rescued by CNIH-2 coexpression, but not by TARP γ-2 coexpression (Fig. 5). Thus, the differential effects between CNIH-2 and other auxiliary subunits do not depend on a particular mutation within the VVLGAVE motif but applies to the AMPAR tetramerization process in general.

### CNIH-2 acts through the AMPAR TMD to promote assembly

**Figure 6:**
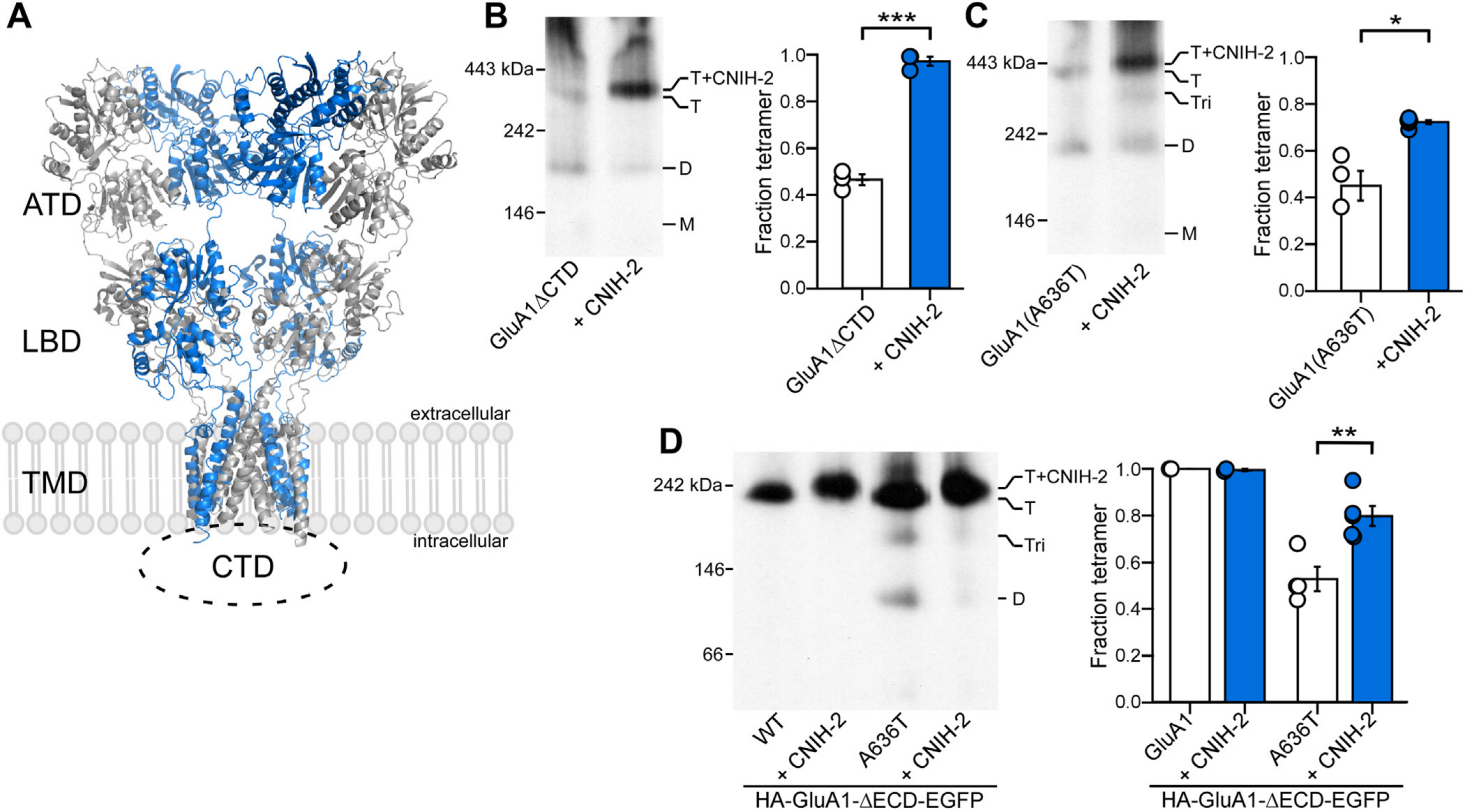
**CNIH-2 acts through the transmembrane domain to promote tetramerization.***A,* structure of AMPARs consist of four modular domains: extracellular amino-terminal (ATD) and ligand-binding (LBD) domains, the transmembrane domain (TMD) forming the ion channel, and the intracellular C-terminal domain (CTD). Homomeric GluA2 tetramer, subunit A′C′ pairs in *gray* and B′D′ pairs in *blue*; PDB: 6XSR (121). *B–D,* BN-PAGE (*left panels*) and normalized tetramer fractions (*right panels*) of GluA1 lacking the C-terminal domain (ΔCTD) (*A*); GluA1 containing the lurcher mutation (A636T) (*B*); and GluA1 lacking the extracellular domains (ΔECD) (*C*). Oligomeric states of the detected bands were estimated by molecular mass and indicated as T (tetramers), Tri (trimers), D (dimers), and M (monomers). Number of samples (*left* to *right*) and significance (*t* test) of tetramer fraction relative to respective control (*white bar*): 3, 3, *p* < 0.001 (B); 4, 5, *p* = 0.013 (*C*); for GluA1: 2, 2, *p* value not determined and for A636T: 4, 5, *p* = 0.007 (*D*). In plots, significance is indicated (*∗p* < 0.05, ∗∗*p* < 0.01, or ∗∗∗*p* < 0.001). AMPAR, α-amino-3-hydroxy-5-methyl-4-isoxazolepropionic acid receptor; BN-PAGE, blue native–PAGE; CNIH, cornichon homolog; ECD, extracellular domain; *ns*, not significant.

Although the M4 segment substitutions disrupt tetramerization and CNIH-2 can rescue these deficits (Figure 2, Figure 3, Figure 4, Figure 5), these results do not indicate that CNIHs interact with the M4 segments to facilitate tetramerization. AMPARs display a modular structure consisting of four major structural domains: extracellular amino-terminal domain (ATD) and ligand-binding domain (LBD), a TMD forming the ion channel, and an intracellular carboxyl-terminal domain (CTD) (Fig. 6*A*). To assess which of these domains mediate the effect of CNIH-2 on AMPAR tetramerization, we used constructs with entire domains deleted from GluA1 (48).

The CTD of AMPAR is involved in receptor trafficking and regulation of receptor functions and AMPAR subunit-dependent (55, 56, 57, 58, 59). The AMPAR CTD plays a critical role in physical interaction with auxiliary subunits and in enabling their modulatory effects on receptor function (60). As found previously, complete deletion of GluA1 (GluA1-ΔCTD) showed a deficit in tetramerization, but this deficit is completely reversed by CNIH-2 coexpression (Fig. 6*B*) (48). Hence, the GluA1 CTD is not required for either the association of CNIH-2 with the receptor or its effect on tetramerization.

Testing the role of the other domains is nontrivial. However, we took an approach to address this question, where the cumulative outcome indicates that CNIH-2 exerts its action *via* the TMD. Initially, we introduced the “lurcher” mutation A636T into GluA1 and coexpressed with CNIH-2. The position, A636, is in the highly conserved “SYTANLAAF” motif of the TMD (Fig. 6*A*) and may disrupt tetramer assembly due to its involvement in the “dimerization of dimers” process (Fig. 6*C*) (61). As shown previously, the lurcher mutation in GluA1 reduces receptor tetramerization (48, 61). Notably, the coexpression of CNIH-2 with the GluA1 lurcher mutant led to a significant increase in the tetramer fraction (Fig. 6*C*), indicating that CNIH-2 can overcome the disruption of the lurcher mutation on tetramerization.

The function of auxiliary subunits has been tied to contacts with the ATD and/or the LBD (62, 63, 64, 65, 66). To evaluate whether the extracellular domains (ECDs), the ATD and the LBD, are required for CNIH-2 to promote GluA1 receptor tetramerization, we used an AMPAR construct lacking the ECDs (GluA1-ΔECD) (48). GluA1-ΔECD efficiently forms tetramers, possibly more efficiently than full-length GluA1 (Fig. 6*D*) (48), making it impossible for CNIH-2 coexpression to further enhance tetramerization. However, there is a slight but notable upshift in molecular mass in the presence of CNIH-2 (Fig. 6*D*), suggesting that the receptor can form a complex with the auxiliary subunit even without its ECDs. To assess whether CNIH-2 enhances the tetrameric stability of the truncated construct, we again took advantage of the lurcher mutation (A636T), which destabilized the tetramer formed by GluA1-ΔECD (Fig. 6*D*). Coexpression of CNIH-2 significantly enhanced the tetrameric fraction, indicating that even without the ECD, CNIH-2 still enhances tetramerization (Fig. 6*D*).

In summary, these results indicate that neither the CTD nor the extracellular ATD and LBD of GluA1 is required for CNIH-2 to enhance receptor tetramerization. Taken together, our experiments suggest that interactions between the TMD of GluA1 and CNIH-2 can promote the process of AMPAR biogenesis. This outcome is consistent with the general structural arrangement of AMPAR and CNIHs (67, 68). These experiments also suggest that the effect of CNIH-2 on receptor assembly is not dependent on mutations within the TMD but rather reflects the stability of the tetrameric complex.

### Direct attachment of TARP γ-2 to GluA1 enhances receptor tetramerization

**Figure 7:**
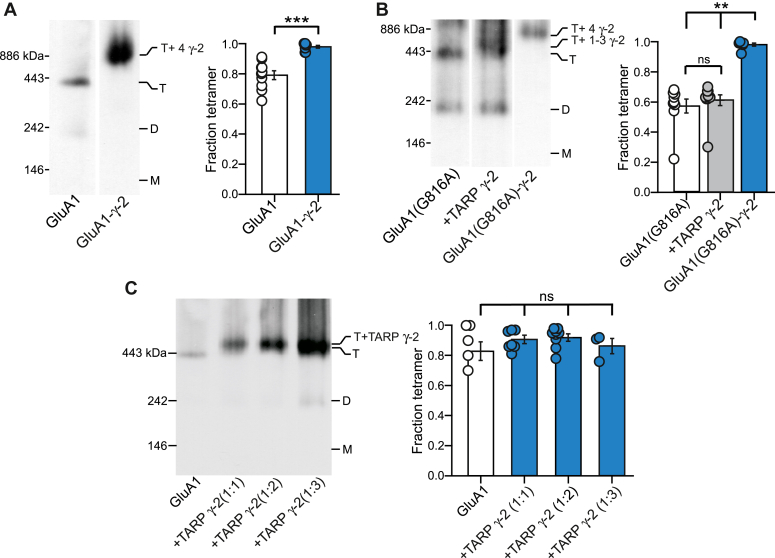
**TARP γ-2 directly attached to GluA1 can enhance tetramerization.***A,* BN-PAGE (*left panel*) and normalized tetramer fractions (*right panel*) of GluA1 or a construct where TARP γ-2 is directly attached to GluA1 (GluA1-γ-2). Number of samples (10 and 8, *left* to *right*) and significance (*t* test) of tetramer fraction relative to respective control (*white bar*): *p* < 0.001. *B,* BN-PAGE (*left panel*) and normalized tetramer fractions (*right panel*) for GluA1(G816A) expressed alone or with TARP γ-2 (+γ-2) or the GluA1-γ-2 tandem construct, where the G816A mutation has been introduced (GluA1(G816A)-γ-2). *C,* BN-PAGE (*left panel*) and normalized tetramer fractions (B) of GluA1 expressed alone or with TARP γ-2 at various DNA ratios (AMPAR subunit: TARP γ-2). Number of samples (9, 10, and 7, *left to right*) and significance (one-way ANOVA): F (2, 23) = 32.6; *(*post hoc Tukey test) G816A *versus* g-2, *p* = 0.72; G816A *versus* tandem, *p* < 0.001; g-2 *versus* tandem, *p* < 0.001 (B). Values from (*left* to *right*) and significance (ANOVA) of tetramer fraction relative to control: 7, 6, 8, and 3; *p* = 0.2282, F = 1.542. In plots, significance is indicated (*∗∗p* < 0.01 or ∗∗∗*p* < 0.001). AMPAR, α-amino-3-hydroxy-5-methyl-4-isoxazolepropionic acid receptor;BN-PAGE, blue native–PAGE; *ns*, not significant; TARP, transmembrane AMPAR regulatory protein.

TARP γ-2 has no effect on AMPAR tetramerization (Figs. 1, 3–5). TARP γ-2 may not be able to stabilize the tetramer or does not interact with GluA1 in the early stages of AMPAR biogenesis and associates later during the AMPAR biogenesis process in comparison to CNIH-2 (23). We therefore took advantage of a construct, where the N terminus of the auxiliary subunit is conjoined to the C terminus of GluA1, referred to as the GluA1-TARP γ-2 tandem (16, 69). The expression of WT GluA1-TARP γ-2 tandem predominantly and more efficiently forms tetramers in comparison to WT GluA1 alone (Fig. 7*A*). To further verify this observation, we introduced the G816A mutation, which disrupts tetramerization (Fig. 2*A*), into the GluA1-TARP γ-2 tandem. As shown previously (Fig. 3*C*), GluA1(G816A) coexpressed with TARP γ-2 alone had no effect on the tetramer fractions. In contrast, GluA1(G816A)-TARP γ-2 tandem showed almost complete tetramerization (Fig. 7*B*).

Additionally, the tetramer band of GluA1(G816A) coexpressed with TARP γ-2 appeared at a lower molecular mass than that of the GluA1(G816A)-TARP γ-2 tandem that is predicted to have a 4:4 stoichiometry (Fig. 7*B*). One possible explanation is that TARP γ-2 have a submaximal stoichiometry (4:1, 2, or 3, AMPAR:γ-2) when coexpressed in human embryonic kidney (HEK) 293 cells at 1:1 ratio, but even at higher DNA concentrations, TARP γ-2 does not increase receptor tetramerization (Fig. 7*C*). Hence, stoichiometry may not be the critical factor but rather it may be the cellular location, where the auxiliary subunit interacts with assembling AMPARs.

### CNIH-2 enhances surface GluA1 expression to a greater extent

To investigate whether the increased tetramerization of GluA1 leads to increased surface trafficking, and consequently higher current amplitudes, we performed whole-cell patch clamp experiments on HEK 293T cells expressing GluA1 alone or with CNIH-2 or TARP γ-2. We were interested in the number of receptors on the membrane and not the effect of the auxiliary subunits on receptor kinetics, so we recorded currents in 15 μM cyclothiazide to minimize desensitization (70, 71). In preliminary experiments, WT GluA1 showed very high current amplitudes making any increase in functional surface receptors with coexpression of auxiliary subunits difficult to identify (not shown). We therefore took advantage of GluA1(G816A), which shows reduced current amplitudes relative to WT GluA1 in the absence of auxiliary subunits, but like GluA1, its tetramerization is enhanced by CNIH-2 but not by TARP γ-2 (Figs. 1 and 3).

**Figure 8:**
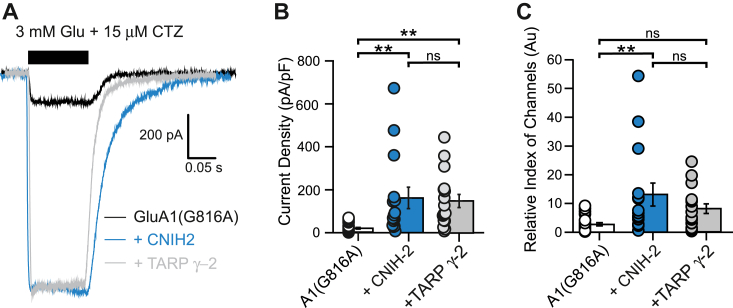
**CNIH-2 enhances GluA1 membrane currents.***A,* whole-cell recordings of GluA1(G816A) expressed alone (*black trace*) or with auxiliary subunits (*blue trace* for CNIH-2; *gray trace* for TARP γ-2) in HEK293T cells. *Solid bar* indicates fast-agonist application of 3 mM glutamate. 15 μM CTZ was present throughout the recording. *B,* quantification (mean ± SEM) of steady-state current amplitudes for GluA1(G816A) expressed alone or with auxiliary subunits (n = 23, 15, 17) normalized to membrane capacitance (C_m_, in pF) as an index of cell size. Significance (one-way ANOVA*):* F(2, 52) = 8.09, *p* = 0.00087; (post hoc Tukey test): G816A *versus* CNIH-2, *p* = 0.0028; G816 A *versus* γ-2, *p* = 0.0057; CNIH-2 *versus* γ-2, *p* = 0.94. *C,* quantification of relative index of channels of GluA1(G816A) expressed alone or with auxiliary subunits (n = 23, 15, 17). See Experimental procedures for normalization process. Significance (one-way ANOVA): F(2,52) = 6.06; (post hoc Tukey test): G816A *versus* CNIH-2, *p* = 0.0032; G816A *versus* γ-2, *p* = 0.16; CNIH-2 *versus* γ-2, *p* = 0.28. Significant differences of relative channel index are indicated (∗∗*p* < 0.01; *ns*, not significant); CNIH, cornichon homolog; CTZ, cyclothiazide; HEK, human embryonic kidney; TARP, transmembrane AMPAR regulatory protein.

GluA1(G816A) showed reduced current amplitudes that were strongly enhanced by CNIH-2 and TARP γ-2 (Fig. 8*A*). In terms of current density, the amplitudes were equally increased for CNIH-2 and TARP γ-2 (Fig. 8*B*). However, TARP γ-2 has a much stronger effect on enhancing GluA1 gating (*P*_*open*_) than CNIH-2 (22). We therefore derived a “relative index of channels” as a measure of density of GluA1 channels on the cell surface as outlined in Experimental procedures (Fig. 8*C*), assuming that auxiliary subunits have the same effect on the channel properties of mutant and WT GluA1 receptors. Based on this index, we found that CNIH-2 significantly enhanced surface expression of GluA1(G816A) relative to the control as well as relative to TARP γ-2. These results support the idea that enhanced receptor tetramerization leads to a functional effect on receptor membrane expression.

**Figure 9:**
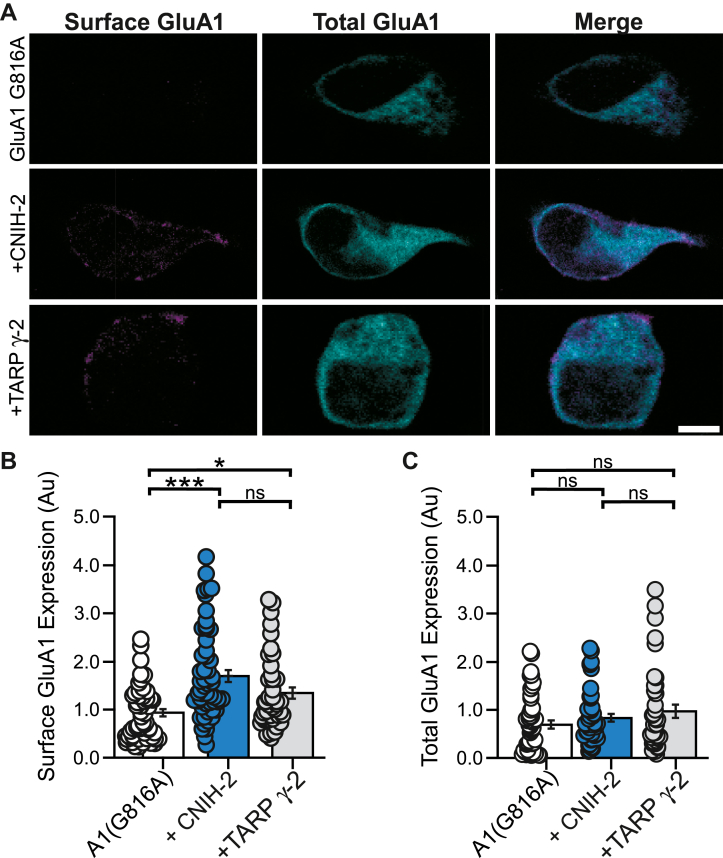
**CNIH-2 enhances GluA1 surface expression.***A,* immunocytochemistry of Neuro2A cells cotransfected with GluA1(G816A)-eGFP alone or with auxiliary subunits, CNIH-2 or TARP γ-2. Surface GluA1 were immunostained using anti-GluA1 under nonpermeabilized conditions. The scale bar represents 5 μM. *B,* quantification (mean ± SEM) of surface GluA1 expressed alone or with auxiliary subunits. Surface expression is normalized to GluA1(G816A) alone. GluA1(G816A), n = 52 cells; GluA1(G816A) + CNIH-2, n = 54 cells; GluA1(G816A) + TARP γ-2, n = 42 cells from three independent experiments. Significance (one-way ANOVA): F(2,145) = 13.2, *p < 0.0001*; (post hoc Tukey test): G816A *versus* CNIH-2, *p* < 0.001; G816A *versus* γ-2, *p* = 0.028; CNIH-2 *versus* γ-2, *p* = 0.068. *C,* quantification of total GluA1-eGFP expressed alone or with auxiliary subunits. Surface expression is normalized to GluA1(G816A) alone. GluA1(G816A), n = 48 cells; GluA1(G816A) + CNIH-2, n = 49 cells; GluA1(G816A) + TARP γ-2, n = 39 cells from three independent experiments. Significance (one-way ANOVA): F(2,133) = 1.83. *p* = 0.16. Significant differences of are indicated (∗*p* < 0.05, ∗∗∗*p* < 0.001; or *ns*, not significant). CNIH, cornichon; TARP, transmembrane AMPAR regulatory protein.

To further assess whether enhanced tetramerization would lead to enhanced surface expression, we transfected Neuro2A cells with GluA1(G816A) tagged with a C-terminal GFP either alone or with auxiliary subunits (Fig. 9*A*). To assay surface expression, we measured integrated fluorescence of AMPARs present on the surface. Coexpression of CNIH-2 increased surface GluA1(G816A) receptors to a greater extent than TARP γ-2 expression (Fig. 9*B*). On the other hand, the total protein level of GluA1(G816A) is not significantly altered by coexpression with either CNIH-2 or TARP γ-2 (Fig. 9*C*). Together, these data indicate that CNIH-2 carries this distinct ability to increase AMPAR tetramers and enhances the forward trafficking and cell surface expression even when tetramer stability is attenuated.

## Discussion

Assembly of AMPARs into a functional tetramer is required for their trafficking to the membrane surface (10, 72, 73, 74). The mechanisms that govern formation of AMPAR heteromers and homomers are not well defined. The preference of heteromeric assembly is thought to predominate, despite the ER retention of edited GluA2(R) and the rapid ER exit of homomeric GluA1 receptors (4, 75). In this study, we find that auxiliary subunits differentially influence homomeric AMPAR tetramerization. Specifically, CNIH homologs, CNIH-2 and CNIH-3, promote tetramerization of GluA1 AMPARs in a heterologous expression system (Fig. 1), whereas for GluA2, this action is specific to CNIH-2 (Fig. 4). While these effects were prominent in WT GluA1 and GluA2, we also took advantage of a subtle mutation in the M4 transmembrane segment that attenuates tetramerization to further verify these outcomes (Figure 2, Figure 3, Figure 4, Figure 5). In contrast, other auxiliary subunits, including TARP γ-2, TARP γ-8, and GSGL-1, do not have any notable effect on receptor tetramerization. Hence, the differential expression of auxiliary subunits and their interaction with receptors in the ER may represent a distinct mechanism to control their availability for membrane expression.

CNIH-2 enhances tetramerization of both GluA1 and GluA2 homomers (Figs. 1 and 4), suggesting that this effect is unlikely to be subunit-specific and applies to AMPARs in general. These results are consistent with the proposed role of CNIHs as ER chaperones based on their evolutionary origin as ER cargo exporters and their ER-Golgi localization in mammalian cells (24, 26, 76). The loss of CNIH-2 leads to a loss of surface AMPARs (23, 24, 26), and CNIH-2 is well known to promote ER exit of AMPARs (21, 23, 24, 25). Despite the small population of GluA1 homomers, these calcium-permeable GluA1 homomeric receptors are critical in neuronal development, synaptic plasticity, and neurological diseases (58, 77, 78, 79, 80, 81, 82, 83, 84, 85). In the present study, we found that CNIHs promote the assembly of homomeric AMPARs including GluA2 homomeric receptors, which traffic to the surface and are functional under certain conditions (3, 77, 86, 87). However, additional experiments are needed to further test this phenomenon in heteromeric receptors since at present there is no method to express only heteromeric AMPARs in contrast to NMDARs (88).

The actions of CNIH-2 and CNIH-3 are not identical. Compared to CNIH-2, CNIH-3 enhances GluA1 tetramerization but appears less effective (Figs. 1 and 3) and has no effect on GluA2 tetramerization (Fig. 4). Knockout of CNIH-2 in excitatory neurons leads to a dramatic loss of surface GluA1-containing AMPARs, potentially consistent with CNIH-2 enhancing GluA1 tetramerization (26, 89). Global KO of CNIH-3 in mice led to overall increases in GluA2-containing receptors in synaptosomes and no changes in GluA1 expression (90). The phenotype of CNIH-3 KO could arise from a compensatory effect of CNIH-2 or the result of sex-related effects on CNIH-3 function (90). Despite the difference in biogenesis, CNIH-2 and CNIH-3 have comparable effects on gating properties (22). It is possible that CNIH homologs have different binding affinities for AMPAR complexes. However, no current work has quantified binding affinities for AMPARs between CNIH homologs, but both CNIH-2/3 interact with AMPARs and have a highly conserved extracellular loop that is necessary and sufficient to modulate AMPAR function (20, 40, 49).

Current structural data suggests CNIH-2 and CNIH-3 despite their sequence similarity demonstrate differences at the M2-N helix, which is an elongated 16-amino acid sequence necessary for maintaining a fully active AMPAR conformation (67, 68). Comparing CNIH-2 and CNIH-3, the M2-N sequence contains subtle differences at the sequence level that could contribute to distinction between CNIHs. In addition to the sequence differences, AMPAR–CNIH complexes also contain lipid-binding sites, which may regulate CNIH function, but also differ in their modulation of CNIH homologs (67, 68). Hence, the basis for the difference in receptor assembly between CNIH-2 and CNIH-3 is unknown but may lie in subtle differences in interactions with AMPARs and the lipid interface.

It is unknown whether tetramerization is a limiting factor of AMPAR trafficking in neurons. It is possible that instead of increasing the overall size of the tetramer pool, CNIHs subtly shift the composition of the tetramers by selectively promoting the assembly of specific AMPAR subunit combinations. Indeed, the trafficking effect of CNIH-2 appears to be splice variant–dependent, and the loss of CNIH-2/3 from hippocampal neurons specifically impacts the surface expression of GluA1-containing receptors (24, 26).

CNIH-2 and CNIH-3 interact with AMPARs *via* the LBD, TMD, and the LBD-TMD linkers of GluA2 (49, 67, 91). Using truncated constructs lacking entire structural domains, we show that neither the ECD (LBD and ATD) nor the intracellular CTD of GluA1 is required for CNIH-2 to enhance tetramerization (Fig. 6). The interface at the transmembrane level of CNIHs and AMPARs are most likely responsible for the ability of CNIHs to promote AMPAR tetramers. Intriguingly, the interaction of the extracellular loop region of CNIHs and the LBD of AMPARs is required for functional modulation but does not seem important for enhanced receptor tetramerization (49). Hence, CNIHs may stabilize certain intersubunit interactions within the AMPAR TMD, such as those between the M4 segment and inner channel core (44). Alternatively, CNIH-2 could exert its effect by interacting with and recruiting components of the AMPAR ER interactome such as FRRS1L, which in turn helps stabilize the tetramer formation in the ER (92, 93).

Glutamate receptor tetramerization is a prerequisite for ER exit (4, 23, 44, 48, 94). The effect of CNIH-2 on initiation of ER exit is in part due to increased availability of fully assembled tetrameric complexes (95). Our experiments, however, suggest that in heterologous cells, the abundance of tetramers in the ER is not a limiting factor for the number of surface-expressed receptors, since coexpression with TARP γ-2 was able to enhance the surface trafficking of GluA1(G816A) without rescuing the defect in tetramerization (Fig. 9*B*). Both CNIH-2 and TARP γ-2 increase the surface expression of receptors in heterologous cells and primary neurons (21, 24, 96). Our experiments in HEK293 cells show that CNIH-2 and TARP γ-2 increase the current amplitudes for exogenously expressed GluA1(G816A) to a similar degree but most likely perform these effects through different mechanisms (Fig. 8, *A* and *B*). The enhancement of current amplitude by TARP γ-2 can be largely accounted for by its ability to boost single-channel conductance and open probability (22, 97, 98). In contrast, since CNIH-2 does not enhance open probability as prominently as TARP γ-2, its effect on current amplitude most likely reflects its ability to promote assembly and surface trafficking of GluA1(G618A) (Figs. 8*C* and 9*B*). We speculate that the main mechanism by which CNIH-2 promotes surface trafficking is to efficiently promote ER exit of fully assembled tetramers to the surface. Accordingly, stepwise expression of CNIH-2 with AMPARs compared to TARP γ-2 is more efficient in promoting assembled receptors (22, 23, 99). The significance of this difference in the context of AMPAR biogenesis suggests that CNIH-2 (100) differs from other auxiliary subunits in the stage at which it alters AMPAR maturation.

In contrast to CNIH-2, TARP γ-2 has little effect on AMPAR tetramerization with or without an EYFP tag (Figure 1, Figure 2, Figure 3 and 6), despite the association of TARP γ-2 with receptors in the ER (30). Instead, a maximal (4:4) stoichiometry and forced association, achieved by expressing GluA1 as a tandem with TARP γ-2, is required for the effect to manifest (Fig. 7). However, increased TARP γ-2 expression (Fig. 7*C*) does not rescue the receptor tetramerization, suggesting that early association in nascent AMPAR complexes in the ER are needed to promote tetramerization. Electrophysiological evidence suggests that AMPAR–TARP complexes display variable stoichiometry depending on neuronal types and the presence of other auxiliary subunits (16, 69, 99, 101), a notion that is also supported by recent structural data of native receptors (53). One possible explanation is that the maximal stoichiometry of 4:4 between GluA1 and TARP γ-2 has a lower incidence and is dependent on expression levels of TARP γ-2 (61, 69, 101). We did increase the DNA ratio of TARP γ-2 to AMPAR subunits to increase overall expression levels, but we did not see a significant increase in GluA1 tetramer formation (Fig. 7*C*), suggesting that factors in addition to stoichiometry may be important (see below).

AMPAR receptors demonstrate strong TARP γ-2 stoichiometry-dependent modulation, which varies across neuronal cell types (16, 69, 102). Similar to previous work, we found clear stoichiometry dependence for TARP γ-2 (Fig. 7). Due to the inherent ambiguity of molecular mass measurement in native gels and the unavailability of a viable GluA1-CNIH-2 tandem constructs, we could not determine whether CNIH-2 coexpressed with GluA1 in our experiments reached a maximal stoichiometry (103). It is possible that the effect of CNIHs on AMPAR tetramerization, unlike that of TARPs, is independent of stoichiometry. Recent structural work with native proteins has added to our knowledge of AMPARs-auxiliary subunits stoichiometry from brain tissue (53, 104). More refined experiments are necessary to clearly define stoichiometry across the AMPAR-auxiliary subunits assemblies.

An alternative explanation of the differential effects between TARP γ-2 coexpression and γ-2 tandem constructs relates to the stage at which TARP γ-2 associates with AMPARs: when coexpressed in HEK293 cells, TARP γ-2 and AMPARs may only interact at later stages of receptor biogenesis (*e.g.,* in Golgi complex). In the stargazer mice, the lack of TARP γ-2 strongly reduces surface AMPARs, but total receptors are only minimally reduced (105). The presence of TARP γ-2 in the ER is also minimal when compared to abundance of CNIH-2 (23). Despite the evidence that TARP γ-2 is in close proximity to AMPARS in the ER and enhances mature glycosylation and surface expression of AMPARs, there is no clear evidence for what stage of assembly that TARP γ-2 associates with AMPARs (30, 105, 106). In contrast, when expressed as a tandem construct, TARP γ-2 and AMPARs are forced to interact in the ER, where AMPAR tetramerization occurs. We speculate that CNIH-2 influences AMPAR tetramerization because of its existence in the receptor complexes in the early stages of receptor biogenesis compared to other auxiliary subunits (23, 24).

In the present work, we have revealed CNIH homologs promote tetramerization of homomeric AMPARs in heterologous cells. Although HEK293 cells have been used extensively in demonstrating functional properties of recombinant AMPARs, there are several limitations including lack of synaptic structures, native posttranslational modifications, and protein overexpression. Future experiments will need to take these experiments into more native environments where endogenous function of AMPAR-auxiliary assemblies can be evaluated. Our experimental system despite limitations in the native environment provides new avenues to testing other nonauxiliary subunits and their association with complexes. Indeed, our use of BN-PAGE comes with caveats including n-dodecyl-β-maltoside (DDM) which could have differential effects on protein associations, which has been demonstrated previously and implicates differences in binding affinities (107, 108). However, with the use of DDM, there is a clear shift in the molecular weight in the AMPAR tetrameric complexes only when auxiliary subunits are expressed, demonstrating that it does not impede auxiliary subunit association with the tetrameric receptors (Figure 1, Figure 2, Figure 4, Figure 5, Figure 6). However, additional work is needed to investigate the number of auxiliary subunits present in these complexes using more quantitative immunoblotting (*e.g.,* infrared conjugated antibodies), which labels individual auxiliary subunits and receptors within the same blot. We also propose the use of methods to evaluate trafficking of different AMPAR complexes and auxiliary subunits using Halotags to differential label surface and intracellular proteins (109, 110).

Auxiliary subunits play a key role in regulating AMPAR function at the surface, including channel gating and synaptic targeting (13). However, prior studies have not investigated how auxiliary subunits may regulate receptor assembly. Our study found that CNIHs can differentially regulate AMPAR assembly by increasing the fraction of tetramers. The early stages of AMPAR biogenesis can be disrupted in pathological conditions (1, 93) and may be crucial to our understanding of neurological disorders including schizophrenia, which show reduced forward trafficking and/or reduction in AMPAR activity (111, 112). Overall, understanding regulation of receptor biogenesis and pathological conditions by auxiliary subunits requires further investigation and is implicated by the genetic risk of psychiatric and addiction susceptibility of *CNIH-2* and *CNIH-3* (113, 114, 115).

## Experimental procedures

### Molecular biology and heterologous expression

AMPAR cDNA constructs are rat (*Rattus norvegicus*) flip splice variants for GluA1 (#P19490) and the edited (R) form of GluA2 (#P19491). Mutations were introduced in these constructs using PCR-based site-directed mutagenesis methods and were validated by DNA sequencing. Numbering is for the mature AMPAR protein with signal peptides: GluA1, 18 residues; GluA2, 21 residues (note: in earlier publications, we did not include the signal peptide). The GluA1-ΔCTD, HA-GluA1-ΔETD-EGFP, and GluA1-EGFP (C terminal) were generated as described previously (44, 48). The cDNA constructs were provided by the following: Dr David Bredt (Johnson & Johnson), TARP γ-2-EYFP; Dr Bernd Fakler (Freiburg), human CNIH-2 (Q6PI25) and GSG1-L (Q6UXU4); Dr Stefan Herlitze (Ruhr), rat TARP γ-2 (Q71RJ2); Dr Roger Nicoll (UCSF), GluA1-TARP γ-2 tandem; and Susumu Tomita (Yale), human CNIH-3 (Q8TBE1) and mouse TARP γ-8 (Q8VHW2). The GluA1 tandem contains GluA1 cojoined at the C terminus with a short linker sequence to the N terminus of rat TARP γ-2 (16).

For BN-PAGE and whole-cells recordings, HEK 293 cells (ATCC, CRL-1573) and HEK 293T (ATCC, CRL-3216) were transiently transfected with cDNAs using Xtreme Gene HP (Roche). For ICC, Neuro-2a (N2a) (ATCC, CCL-131) were transiently transfected with cDNAs using Xtreme Gene HP (Roche). AMPARs and auxiliary subunits were cotransfected at a cDNA ratio of 1:1. Transfected cells were maintained in media containing 5% fetal bovine serum supplemented with 10 μM CNQX (Sigma) and 10 μM NBQX (Sigma) to minimize AMPAR-mediated excitotoxicity (116).

### Blue native–PAGE

HEK 293 cells were plated on 60 mm tissue culture dishes and transfected 24 h later at 70% confluency. From 30 to 48 h after transfection, the cells were harvested as previously described (117). Membrane proteins extracted from whole-cell lysates were resolved using BN-PAGE as previously described (48, 103, 117). Briefly, protein samples mixed with 1× native PAGE sample buffer, 0.05% native PAGE G-250 additive, and 10 mMn-DDM were loaded onto Novex 4 to 16% Bis/Tris gradient gels (Life Technologies). When assaying stoichiometry of AMPAR-auxiliary subunit complexes, 3 to 12% Bis-tris gels were used to better resolve protein complexes at higher molecular masses. Molecular mass markers were also loaded and included NativeMark Unstained Protein Standard (Life Technologies) and Apoferritin (Sigma). BN-PAGE and transfer were conducted as previously described (48, 117). BN-PAGE samples were analyzed by Western blot. Anti-GluA1 (Millipore, MAB2269, 1:1500) and Anti-GluA2 (Millipore; MAB297, 1:500) mouse mAbs were used to detect specific AMPAR subunits. HRP-conjugated anti-mouse immunoglobulin G (Santa Cruz Biotechnologies, sc-2031, 1:1000) was used as a secondary antibody. Blots were developed using luminol reagent (Santa Cruz Biotechnologies, sc-2048) before exposure to chemiluminescence blue–sensitive film. For clarity of presentation, reorganized lanes from the same gel are indicated by a thin space between lanes.

### BN-PAGE densitometry and quantification

Developed immunoblot films were scanned using an EPSON flat scanner (Epson Perfection V700 Photo) into.tiff format in an 8-bit gray scale mode, 300 dpi resolution, and quantified using NIH Image J (https://imagej.nih.gov/ij/) (118). The following parameters including antibody concentration, duration of immunostaining, and time of exposure were optimized to ensure major bands fall within the linear response range of the film (117). Quantification of each resolved band for any oligomeric state (monomer, dimer, and tetramer bands) of AMPARs is based on the relative position to the molecular weight markers. Using ImageJ, the relative measurement of the fraction of tetramer was calculated from the cumulative intensities of the tetramer band(s), which included nonshifted and shifted bands, divided by the cumulative intensities of all oligomers (monomer, dimers, and tetramers) previously described (48, 117). The tetramer fraction data was normalized to 1. The intensity of each band is not necessarily proportional to the total amount of protein within each band because primary antibodies are not guaranteed to bind every single AMPAR subunit in an oligomeric complex. The fraction of tetramer quantification is therefore a relative measurement of tetramerization efficiency and cannot calculate absolute physical quantities as previously shown (103). Comparisons were made within the same cell passage due to variation in gene expression depending on the passage number.

### Whole-cell current recordings

HEK 293T cells were plated in 24-well plates at a density of 4 × 10^5^ cells on uncoated coverslips. Cells were maintained in 10% fetal bovine serum at 37 °C and 95% O_2_/5% CO_2_. AMPAR-mediated currents in transfected HEK 293T cells were recorded 24 to 48 h following transfection. On each recording day, we recorded cells transfected with GluA1, GluA1 + CNIH-2, and GluA1 + TARP γ-2. Recordings were performed in the whole-cell configuration at room temperature (20–23 °C) using an EPC-10 amplifier and Patchmaster software (HEKA Elektronik) (https://www.heka.com/). Signals were low pass filtered at 5 kHz and digitized at 20 kHz. Patch microelectrodes were filled with a KCl-based intracellular solution (in mM): 140 KCl, 2 NaCl, 4 Mg^2+^-ATP, 0.3 Na^+^ -ATP, 1 BAPTA, 10 Hepes, pH 7.2 (KOH), 300 mOsm (sucrose) and had resistances of 4 to 6 MΩ. We did not use series resistance compensation nor did we correct for junction potentials. External solution consisted of the following (in mM): 140 NaCl, 1.8 CaCl_2_, 1 MgCl_2_, and 10 Hepes, pH = 7.2 (NaOH). All solutions contained 15 mM of cyclothiazide to minimize the impact of desensitization on current amplitudes. Glutamate was applied using a piezo-driven double barrel application system with one barrel containing external solution and the other containing the same solution with added glutamate (3 mM) (119, 120). The 10 to 90% rise time of the application pipet was approximately 500 to 800 μs.

We measured membrane capacitance (*C*_*m*_), maximum current amplitudes (*I*_*peak*_), and the current-voltage relationship. The current amplitude is a function of the number of ion channels on the membrane (*N*), the probability of opening (*P*_*open*_), and their single-channel conductance (γ):

$$I_{peak}=N\times P_{open}\times \gamma \times \left(E_{m}-E_{rev}\right)$$

where membrane potential (*E*_*m*_) and reversal potential (*E*_*rev*_) are assumed to be invariant in our experiment. CNIH-2 and TARP γ-2 have differential effects on *P*_*open*_ and γ (22). Since we were interested in comparing the effect of CNIH-2 and TARP γ-2 on the number of channels on the membrane, we calculated *N* using our current amplitudes and previously published values for *P*_*open*_ and γ using the relationship:

$$N=\frac{\frac{I_{peak}}{C_{m}}}{P_{open}\times \gamma }$$

Values for *P*_*open*_ and γ were 0.46 and 30.2 pS (GluA1 + CNIH-2) and 0.64 and 28.2 pS (GluA1 + TARP γ-2), while the values for WT GluA1 alone were 0.41 and 18.7 pS (22). We do not view the derived *N* as absolute but rather as a relative index of channels.

### Immunocytochemistry

N2a cells were transfected with GluA1(G816A)-eGFP (C terminal) and auxiliary subunits at a 1:1 DNA ratio. Post 48-h transfection, transfected N2a cells were stained with anti-GluA1 antibody (MilliporeSigma #MAB2263; 1:500) in conditioned media for 30 min at 37 °C. Coverslips were washed with 1× PBS and then fixed for 10 min with 4% paraformaldehyde and 4% sucrose in 1× PBS, pH 7.4. The coverslips were incubated with blocking solution (5% normal goat serum in 1× PBS) for 1 h at 4 °C. Coverslips were washed with PBS and then incubated with Alexa 633–conjugated goat anti-mouse secondary antibody (Invitrogen #A21052; 1:1500) for 1 h at 4 °C. After washing with PBS, coverslips were stained with NucBlue Ready Probes (Invitrogen #R37606) for 20 min and mounted in Prolong Diamond mount (Invitrogen #P36961).

Images were acquired on an Olympus FV-1000 confocal microscope with an oil-immersion objective (60×, numerical aperture 1.42) using a Fluoview software (FV10-ASW, version 4.02, Olympus) (https://www.olympus-lifescience.com/en/). For each coverslip, 3 to 5 fields were imaged with consistent filter settings. For quantification of surface AMPARs, corrected total cell fluorescence was determined on ImageJ (NIH) by measuring the surface AMPARs (Alexa 633) or total AMPARs (GFP tag) integrated density for the N2a cell area and subtracting off-cell background mean intensity. Intensity was then normalized to the mean intensity of GluA1(G816A) when expressed alone. Quantification, imaging, and image analysis were done blind to treatment conditions. Images collected were blinded using Blind Analysis Tools, an ImageJ plugin.

### Statistics

Data analysis for BN-PAGE, whole-cell recordings, and immunocytochemistry were performed using Igor Pro (version 7, Wave Metrics) (https://www.wavemetrics.com/), Microsoft Excel Analysis ToolPak, and GraphPad Prism 9 (https://www.graphpad.com/). All data values are represented as the mean ± SEM. To ensure reproducibility of results, at least three independent experiments were performed. Analyses of tetramer fraction, current amplitudes, relative channel index, and fluorescence intensity were performed between different constructs or combinations of coexpression using Student’s *t* test or a one-way ANOVA with a post hoc Tukey’s test.

## Data availability

Datasets generated during and/or analyzed during the current study are available from the corresponding author on reasonable request.

## Conflict of interest

The authors declare that they have no conflicts of interest with the contents of this article.

## References

[1] Groc L., Choquet D. (2020). Linking glutamate receptor movements and synapse function. Science.

[2] Hansen K.B., Wollmuth L.P., Bowie D., Furukawa H., Menniti F.S., Sobolevsky A.I. (2021). Structure, function, and pharmacology of glutamate receptor ion channels. Pharmacol. Rev..

[3] Shi S., Hayashi Y., Esteban J.A., Malinow R. (2001). Subunit-specific rules governing AMPA receptor trafficking to synapses in hippocampal pyramidal neurons. Cell.

[4] Greger I.H., Khatri L., Kong X., Ziff E.B. (2003). AMPA receptor tetramerization is mediated by Q/R editing. Neuron.

[5] Lu W., Roche K.W. (2012). Posttranslational regulation of AMPA receptor trafficking and function. Curr. Opin. Neurobiol..

[6] Kandel M.B., Yamamoto S., Midorikawa R., Morise J., Wakazono Y., Oka S. (2018). N-glycosylation of the AMPA-type glutamate receptor regulates cell surface expression and tetramer formation affecting channel function. J. Neurochem..

[7] Jackson A.C., Nicoll R.A. (2011). The expanding social network of ionotropic glutamate receptors: TARPs and other transmembrane auxiliary subunits. Neuron.

[8] Yan D., Tomita S. (2012). Defined criteria for auxiliary subunits of glutamate receptors. J. Physiol..

[9] Kamalova A., Nakagawa T. (2021). AMPA receptor structure and auxiliary subunits. J. Physiol..

[10] Jacobi E., von Engelhardt J. (2021). Modulation of information processing by AMPA receptor auxiliary subunits. J. Physiol..

[11] Greger I.H., Esteban J.A. (2007). AMPA receptor biogenesis and trafficking. Curr. Opin. Neurobiol..

[12] Haering S.C., Tapken D., Pahl S., Hollmann M. (2014). Auxiliary subunits: shepherding AMPA receptors to the plasma membrane. Membranes (Basel).

[13] Bissen D., Foss F., Acker-Palmer A. (2019). AMPA receptors and their minions: auxiliary proteins in AMPA receptor trafficking. Cell Mol. Life Sci..

[14] Sumioka A. (2013). Auxiliary subunits provide new insights into regulation of AMPA receptor trafficking. J. Biochem..

[15] Khodosevich K., Jacobi E., Farrow P., Schulmann A., Rusu A., Zhang L. (2014). Coexpressed auxiliary subunits exhibit distinct modulatory profiles on AMPA receptor function. Neuron.

[16] Shi Y., Lu W., Milstein A.D., Nicoll R.A. (2009). The stoichiometry of AMPA receptors and TARPs varies by neuronal cell type. Neuron.

[17] Schwenk J., Baehrens D., Haupt A., Bildl W., Boudkkazi S., Roeper J. (2014). Regional diversity and developmental dynamics of the AMPA-receptor proteome in the mammalian brain. Neuron.

[18] McGee T.P., Bats C., Farrant M., Cull-Candy S.G. (2015). Auxiliary subunit GSG1L acts to suppress calcium-permeable AMPA receptor function. J. Neurosci..

[19] Jacobi E., von Engelhardt J. (2017). Diversity in AMPA receptor complexes in the brain. Curr. Opin. Neurobiol..

[20] Schwenk J., Harmel N., Zolles G., Bildl W., Kulik A., Heimrich B. (2009). Functional proteomics identify cornichon proteins as auxiliary subunits of AMPA receptors. Science.

[21] Shi Y., Suh Y.H., Milstein A.D., Isozaki K., Schmid S.M., Roche K.W. (2010). Functional comparison of the effects of TARPs and cornichons on AMPA receptor trafficking and gating. Proc. Natl. Acad. Sci. U. S. A..

[22] Coombs I.D., Soto D., Zonouzi M., Renzi M., Shelley C., Farrant M. (2012). Cornichons modify channel properties of recombinant and glial AMPA receptors. J. Neurosci..

[23] Schwenk J., Boudkkazi S., Kocylowski M.K., Brechet A., Zolles G., Bus T. (2019). An ER assembly line of AMPA-receptors controls excitatory neurotransmission and its plasticity. Neuron.

[24] Harmel N., Cokic B., Zolles G., Berkefeld H., Mauric V., Fakler B. (2012). AMPA receptors commandeer an ancient cargo exporter for use as an auxiliary subunit for signaling. PLoS One.

[25] Brockie P.J., Jensen M., Mellem J.E., Jensen E., Yamasaki T., Wang R. (2013). Cornichons control ER export of AMPA receptors to regulate synaptic excitability. Neuron.

[26] Herring B.E., Shi Y., Suh Y.H., Zheng C.Y., Blankenship S.M., Roche K.W. (2013). Cornichon proteins determine the subunit composition of synaptic AMPA receptors. Neuron.

[27] Priel A., Kolleker A., Ayalon G., Gillor M., Osten P., Stern-Bach Y. (2005). Stargazin reduces desensitization and slows deactivation of the AMPA-type glutamate receptors. J. Neurosci..

[28] Cho C.H., St-Gelais F., Zhang W., Tomita S., Howe J.R. (2007). Two families of TARP isoforms that have distinct effects on the kinetic properties of AMPA receptors and synaptic currents. Neuron.

[29] Milstein A.D., Zhou W., Karimzadegan S., Bredt D.S., Nicoll R.A. (2007). TARP subtypes differentially and dose-dependently control synaptic AMPA receptor gating. Neuron.

[30] Bedoukian M.A., Weeks A.M., Partin K.M. (2006). Different domains of the AMPA receptor direct stargazin-mediated trafficking and stargazin-mediated modulation of kinetics. J. Biol. Chem..

[31] Matsuda S., Kakegawa W., Budisantoso T., Nomura T., Kohda K., Yuzaki M. (2013). Stargazin regulates AMPA receptor trafficking through adaptor protein complexes during long-term depression. Nat. Commun..

[32] Constals A., Andrew, Compans B., Toulmé E., Phillipat A., Marais S. (2015). Glutamate-induced AMPA receptor desensitization increases their mobility and modulates short-term plasticity through unbinding from stargazin. Neuron.

[33] Vandenberghe W., Nicoll R.A., Bredt D.S. (2005). Interaction with the unfolded protein response reveals a role for stargazin in biosynthetic AMPA receptor transport. J. Neurosci..

[34] Shanks N.F., Maruo T., Farina A.N., Ellisman M.H., Nakagawa T. (2010). Contribution of the global subunit structure and stargazin on the maturation of AMPA receptors. J. Neurosci..

[35] Chen L., Chetkovich D.M., Petralia R.S., Sweeney N.T., Kawasaki Y., Wenthold R.J. (2000). Stargazin regulates synaptic targeting of AMPA receptors by two distinct mechanisms. Nature.

[36] Kato A.S., Gill M.B., Yu H., Nisenbaum E.S., Bredt D.S. (2010). TARPs differentially decorate AMPA receptors to specify neuropharmacology. Trends Neurosci..

[37] Rouach N., Byrd K., Petralia R.S., Elias G.M., Adesnik H., Tomita S. (2005). TARP gamma-8 controls hippocampal AMPA receptor number, distribution and synaptic plasticity. Nat. Neurosci..

[38] Sumioka A., Brown T.E., Kato A.S., Bredt D.S., Kauer J.A., Tomita S. (2011). PDZ binding of TARPgamma-8 controls synaptic transmission but not synaptic plasticity. Nat. Neurosci..

[39] Zheng C.Y., Chang K., Suh Y.H., Roche K.W. (2015). TARP gamma-8 glycosylation regulates the surface expression of AMPA receptors. Biochem J.

[40] Shanks N.F., Savas J.N., Maruo T., Cais O., Hirao A., Oe S. (2012). Differences in AMPA and kainate receptor interactomes facilitate identification of AMPA receptor auxiliary subunit GSG1L. Cell Rep..

[41] Twomey E.C., Yelshanskaya M.V., Grassucci R.A., Frank J., Sobolevsky A.I. (2017). Structural bases of desensitization in AMPA receptor-auxiliary subunit complexes. Neuron.

[42] Gu X., Mao X., Lussier M.P., Hutchison M.A., Zhou L., Hamra F.K. (2016). GSG1L suppresses AMPA receptor-mediated synaptic transmission and uniquely modulates AMPA receptor kinetics in hippocampal neurons. Nat. Commun..

[43] Kamalova A., Futai K., Delpire E., Nakagawa T. (2020). AMPA receptor auxiliary subunit GSG1L suppresses short-term facilitation in corticothalamic synapses and determines seizure susceptibility. Cell Rep..

[44] Salussolia C.L., Gan Q., Kazi R., Singh P., Allopenna J., Furukawa H. (2013). A eukaryotic specific transmembrane segment is required for tetramerization in AMPA receptors. J. Neurosci..

[45] Morise J., Suzuki K.G.N., Kitagawa A., Wakazono Y., Takamiya K., Tsunoyama T.A. (2019). AMPA receptors in the synapse turnover by monomer diffusion. Nat. Commun..

[46] Penn A.C., Williams S.R., Greger I.H. (2008). Gating motions underlie AMPA receptor secretion from the endoplasmic reticulum. EMBO J..

[47] Rossmann M., Sukumaran M., Penn A.C., Veprintsev D.B., Babu M.M., Greger I.H. (2011). Subunit-selective N-terminal domain associations organize the formation of AMPA receptor heteromers. EMBO J..

[48] Gan Q., Dai J., Zhou H.X., Wollmuth L.P. (2016). The transmembrane domain mediates tetramerization of alpha-Amino-3-hydroxy-5-methyl-4-isoxazolepropionic acid (AMPA) receptors. J. Biol. Chem..

[49] Shanks N.F., Cais O., Maruo T., Savas J.N., Zaika E.I., Azumaya C.M. (2014). Molecular dissection of the interaction between the AMPA receptor and cornichon homolog-3. J. Neurosci..

[50] Amin J.B., Salussolia C.L., Chan K., Regan M.C., Dai J., Zhou H.X. (2017). Divergent roles of a peripheral transmembrane segment in AMPA and NMDA receptors. J. Gen. Physiol..

[51] Salussolia C.L., Corrales A., Talukder I., Kazi R., Akgul G., Bowen M. (2011). Interaction of the M4 segment with other transmembrane segments is required for surface expression of mammalian alpha-amino-3-hydroxy-5-methyl-4-isoxazolepropionic acid (AMPA) receptors. J. Biol. Chem..

[52] Sobolevsky A.I., Rosconi M.P., Gouaux E. (2009). X-ray structure, symmetry and mechanism of an AMPA-subtype glutamate receptor. Nature.

[53] Zhao Y., Chen S., Swensen A.C., Qian W.J., Gouaux E. (2019). Architecture and subunit arrangement of native AMPA receptors elucidated by cryo-EM. Science.

[54] Pick J.E., Ziff E.B. (2018). Regulation of AMPA receptor trafficking and exit from the endoplasmic reticulum. Mol. Cell Neurosci..

[55] Tomita S., Adesnik H., Sekiguchi M., Zhang W., Wada K., Howe J.R. (2005). Stargazin modulates AMPA receptor gating and trafficking by distinct domains. Nature.

[56] Granger A.J., Shi Y., Lu W., Cerpas M., Nicoll R.A. (2013). LTP requires a reserve pool of glutamate receptors independent of subunit type. Nature.

[57] Jenkins M.A., Wells G., Bachman J., Snyder J.P., Jenkins A., Huganir R.L. (2014). Regulation of GluA1 α-amino-3-hydroxy-5-methyl-4-isoxazolepropionic acid receptor function by protein kinase C at serine-818 and threonine-840. Mol. Pharmacol..

[58] Zhou Z., Liu A., Xia S., Leung C., Qi J., Meng Y. (2018). The C-terminal tails of endogenous GluA1 and GluA2 differentially contribute to hippocampal synaptic plasticity and learning. Nat. Neurosci..

[59] Diaz-Alonso J., Morishita W., Incontro S., Simms J., Holtzman J., Gill M. (2020). Long-term potentiation is independent of the C-tail of the GluA1 AMPA receptor subunit. Elife.

[60] Ben-Yaacov A., Gillor M., Haham T., Parsai A., Qneibi M., Stern-Bach Y. (2017). Molecular mechanism of AMPA receptor modulation by TARP/stargazin. Neuron.

[61] Kim K.S., Yan D., Tomita S. (2010). Assembly and stoichiometry of the AMPA receptor and transmembrane AMPA receptor regulatory protein complex. J. Neurosci..

[62] Cais O., Herguedas B., Krol K., Cull-Candy S.G., Farrant M., Greger I.H. (2014). Mapping the interaction sites between AMPA receptors and TARPs reveals a role for the receptor N-terminal domain in channel gating. Cell Rep..

[63] Riva I., Eibl C., Volkmer R., Carbone A.L., Plested A.J. (2017). Control of AMPA receptor activity by the extracellular loops of auxiliary proteins. Elife.

[64] Watson J.F., Pinggera A., Ho H., Greger I.H. (2021). AMPA receptor anchoring at CA1 synapses is determined by N-terminal domain and TARP gamma8 interactions. Nat. Commun..

[65] Klykov O., Gangwar S.P., Yelshanskaya M.V., Yen L., Sobolevsky A.I. (2021). Structure and desensitization of AMPA receptor complexes with type II TARP gamma5 and GSG1L. Mol. Cell.

[66] Herguedas B., Kohegyi B.K., Dohrke J.N., Watson J.F., Zhang D., Ho H. (2022). Mechanisms underlying TARP modulation of the GluA1/2-gamma8 AMPA receptor. Nat. Commun..

[67] Nakagawa T. (2019). Structures of the AMPA receptor in complex with its auxiliary subunit cornichon. Science.

[68] Zhang D., Watson J.F., Matthews P.M., Cais O., Greger I.H. (2021). Gating and modulation of a hetero-octameric AMPA glutamate receptor. Nature.

[69] Miguez-Cabello F., Sanchez-Fernandez N., Yefimenko N., Gasull X., Gratacos-Batlle E., Soto D. (2020). AMPAR/TARP stoichiometry differentially modulates channel properties. Elife.

[70] Sun Y., Olson R., Horning M., Armstrong N., Mayer M., Gouaux E. (2002). Mechanism of glutamate receptor desensitization. Nature.

[71] Milstein A.D., Nicoll R.A. (2008). Regulation of AMPA receptor gating and pharmacology by TARP auxiliary subunits. Trends Pharmacol. Sci..

[72] Gan Q., Salussolia C.L., Wollmuth L.P. (2015). Assembly of AMPA receptors: mechanisms and regulation. J. Physiol..

[73] Greger I.H., Watson J.F., Cull-Candy S.G. (2017). Structural and functional architecture of AMPA-type glutamate receptors and their auxiliary proteins. Neuron.

[74] Schwenk J., Fakler B. (2021). Building of AMPA-type glutamate receptors in the endoplasmic reticulum and its implication for excitatory neurotransmission. J. Physiol..

[75] Mansour M., Nagarajan N., Nehring R.B., Clements J.D., Rosenmund C. (2001). Heteromeric AMPA receptors assemble with a preferred subunit stoichiometry and spatial arrangement. Neuron.

[76] Bokel C., Dass S., Wilsch-Brauninger M., Roth S. (2006). Drosophila Cornichon acts as cargo receptor for ER export of the TGFalpha-like growth factor Gurken. Development.

[77] Wenthold R.J., Petralia R.S., Blahos J., Niedzielski A.S. (1996). Evidence for multiple AMPA receptor complexes in hippocampal CA1/CA2 neurons. J. Neurosci..

[78] Conrad K.L., Tseng K.Y., Uejima J.L., Reimers J.M., Heng L.J., Shaham Y. (2008). Formation of accumbens GluR2-lacking AMPA receptors mediates incubation of cocaine craving. Nature.

[79] Brill J., Huguenard J.R. (2008). Sequential changes in AMPA receptor targeting in the developing neocortical excitatory circuit. J. Neurosci..

[80] Kessels H.W., Malinow R. (2009). Synaptic AMPA receptor plasticity and behavior. Neuron.

[81] McCutcheon J.E., Wang X., Tseng K.Y., Wolf M.E., Marinelli M. (2011). Calcium-permeable AMPA receptors are present in nucleus accumbens synapses after prolonged withdrawal from cocaine self-administration but not experimenter-administered cocaine. J. Neurosci..

[82] Ge Y., Tian M., Liu L., Wong T.P., Gong B., Wu D. (2019). p97 regulates GluA1 homomeric AMPA receptor formation and plasma membrane expression. Nat. Commun..

[83] Ismail V., Zachariassen L.G., Godwin A., Sahakian M., Ellard S., Stals K.L. (2022). Identification and functional evaluation of GRIA1 missense and truncation variants in individuals with ID: an emerging neurodevelopmental syndrome. Am. J. Hum. Genet..

[84] Azarnia Tehran D., Kochlamazashvili G., Pampaloni N.P., Sposini S., Shergill J.K., Lehmann M. (2022). Selective endocytosis of Ca(2+)-permeable AMPARs by the Alzheimer's disease risk factor CALM bidirectionally controls synaptic plasticity. Sci. Adv..

[85] Coombs I., Bats C., Sexton C.A., Studniarczyk D., Cull-Candy S.G., Farrant M. (2023). Enhanced functional detection of synaptic calcium-permeable AMPA receptors using intracellular NASPM. Elife.

[86] Lu W., Shi Y., Jackson A.C., Bjorgan K., During M.J., Sprengel R. (2009). Subunit composition of synaptic AMPA receptors revealed by a single-cell genetic approach. Neuron.

[87] Coombs I.D., Soto D., McGee T.P., Gold M.G., Farrant M., Cull-Candy S.G. (2019). Homomeric GluA2(R) AMPA receptors can conduct when desensitized. Nat. Commun..

[88] Hansen K.B., Ogden K.K., Yuan H., Traynelis S.F. (2014). Distinct functional and pharmacological properties of Triheteromeric GluN1/GluN2A/GluN2B NMDA receptors. Neuron.

[89] Liu M., Shi R., Hwang H., Han K.S., Wong M.H., Ren X. (2018). SAP102 regulates synaptic AMPAR function through a CNIH-2-dependent mechanism. J. Neurophysiol..

[90] Frye H.E., Izumi Y., Harris A.N., Williams S.B., Trousdale C.R., Sun M.Y. (2021). Sex differences in the role of CNIH3 on spatial memory and synaptic plasticity. Biol. Psychiatry.

[91] Hawken N.M., Zaika E.I., Nakagawa T. (2017). Engineering defined membrane-embedded elements of AMPA receptor induces opposing gating modulation by cornichon 3 and stargazin. J. Physiol..

[92] Rubio M.E., Wenthold R.J. (1999). Calnexin and the immunoglobulin binding protein (BiP) coimmunoprecipitate with AMPA receptors. J. Neurochem..

[93] Brechet A., Buchert R., Schwenk J., Boudkkazi S., Zolles G., Siquier-Pernet K. (2017). AMPA-receptor specific biogenesis complexes control synaptic transmission and intellectual ability. Nat. Commun..

[94] Nakagawa T. (2010). The biochemistry, ultrastructure, and subunit assembly mechanism of AMPA receptors. Mol. Neurobiol..

[95] Fleck M.W. (2006). Glutamate receptors and endoplasmic reticulum quality control: looking beneath the surface. Neuroscientist.

[96] Kato A.S., Gill M.B., Ho M.T., Yu H., Tu Y., Siuda E.R. (2010). Hippocampal AMPA receptor gating controlled by both TARP and cornichon proteins. Neuron.

[97] Shelley C., Farrant M., Cull-Candy S.G. (2012). TARP-associated AMPA receptors display an increased maximum channel conductance and multiple kinetically distinct open states. J. Physiol..

[98] Soto D., Altafaj X., Sindreu C., Bayes A. (2014). Glutamate receptor mutations in psychiatric and neurodevelopmental disorders. Commun. Integr. Biol..

[99] Gill M.B., Kato A.S., Roberts M.F., Yu H., Wang H., Tomita S. (2011). Cornichon-2 modulates AMPA receptor-transmembrane AMPA receptor regulatory protein assembly to dictate gating and pharmacology. J. Neurosci..

[100] Erlenhardt N., Yu H., Abiraman K., Yamasaki T., Wadiche J.I., Tomita S. (2016). Porcupine controls hippocampal AMPAR levels, composition, and synaptic transmission. Cell Rep..

[101] Hastie P., Ulbrich M.H., Wang H.L., Arant R.J., Lau A.G., Zhang Z. (2013). AMPA receptor/TARP stoichiometry visualized by single-molecule subunit counting. Proc. Natl. Acad. Sci. U. S. A..

[102] Dawe G.B., Kadir M.F., Venskutonyte R., Perozzo A.M., Yan Y., Alexander R.P.D. (2019). Nanoscale mobility of the apo state and TARP stoichiometry dictate the gating behavior of alternatively spliced AMPA receptors. Neuron.

[103] Schagger H., Cramer W.A., von Jagow G. (1994). Analysis of molecular masses and oligomeric states of protein complexes by blue native electrophoresis and isolation of membrane protein complexes by two-dimensional native electrophoresis. Anal. Biochem..

[104] Yu J., Rao P., Clark S., Mitra J., Ha T., Gouaux E. (2021). Hippocampal AMPA receptor assemblies and mechanism of allosteric inhibition. Nature.

[105] Tomita S., Chen L., Kawasaki Y., Petralia R.S., Wenthold R.J., Nicoll R.A. (2003). Functional studies and distribution define a family of transmembrane AMPA receptor regulatory proteins. J. Cell Biol..

[106] Vandenberghe W., Nicoll R.A., Bredt D.S. (2005). Stargazin is an AMPA receptor auxiliary subunit. Proc. Natl. Acad. Sci. U. S. A..

[107] Nakagawa T., Cheng Y., Ramm E., Sheng M., Walz T. (2005). Structure and different conformational states of native AMPA receptor complexes. Nature.

[108] Nakagawa T., Cheng Y., Sheng M., Walz T. (2006). Three-dimensional structure of an AMPA receptor without associated stargazin/TARP proteins. Biol. Chem..

[109] Los G.V., Encell L.P., McDougall M.G., Hartzell D.D., Karassina N., Zimprich C. (2008). HaloTag: a novel protein labeling technology for cell imaging and protein analysis. ACS Chem. Biol..

[110] Watson E.T., Pauers M.M., Seibert M.J., Vevea J.D., Chapman E.R. (2023). Synaptic vesicle proteins are selectively delivered to axons in mammalian neurons. Elife.

[111] Zeppillo T., Schulmann A., Macciardi F., Hjelm B.E., Focking M., Sequeira P.A. (2020). Functional impairment of cortical AMPA receptors in schizophrenia. Schizophr. Res..

[112] Yonezawa K., Tani H., Nakajima S., Nagai N., Koizumi T., Miyazaki T. (2022). AMPA receptors in schizophrenia: a systematic review of postmortem studies on receptor subunit expression and binding. Schizophr. Res..

[113] Drummond J.B., Simmons M., Haroutunian V., Meador-Woodruff J.H. (2012). Upregulation of cornichon transcripts in the dorsolateral prefrontal cortex in schizophrenia. Neuroreport.

[114] Nelson E.C., Agrawal A., Heath A.C., Bogdan R., Sherva R., Zhang B. (2016). Evidence of CNIH3 involvement in opioid dependence. Mol. Psych..

[115] Han S., Lin Y., Wang M., Goes F.S., Tan K., Zandi P. (2018). Integrating brain methylome with GWAS for psychiatric risk gene discovery. bioRxiv.

[116] Menuz K., Stroud R.M., Nicoll R.A., Hays F.A. (2007). TARP auxiliary subunits switch AMPA receptor antagonists into partial agonists. Science.

[117] Salussolia C.S., Gan G., Wollmuth L.P., Popescu G.K. (2016). Neuromethods.

[118] Schneider C.A., Rasband W.S., Eliceiri K.W. (2012). NIH Image to ImageJ: 25 years of image analysis. Nat. Methods.

[119] Yelshansky M.V., Sobolevsky A.I., Jatzke C., Wollmuth L.P. (2004). Block of AMPA receptor desensitization by a point mutation outside the ligand-binding domain. J. Neurosci..

[120] Amin J.B., Leng X., Gochman A., Zhou H.X., Wollmuth L.P. (2018). A conserved glycine harboring disease-associated mutations permits NMDA receptor slow deactivation and high Ca(2+) permeability. Nat. Commun..

[121] Yelshanskaya M.V., Singh A.K., Narangoda C., Williams R.S.B., Kurnikova M.G., Sobolevsky A.I. (2022). Structural basis of AMPA receptor inhibition by trans-4-butylcyclohexane carboxylic acid. Br. J. Pharmacol..

